# High-speed and high-SNR photoacoustic microscopy based on a galvanometer mirror in non-conducting liquid

**DOI:** 10.1038/srep34803

**Published:** 2016-10-06

**Authors:** Jin Young Kim, Changho Lee, Kyungjin Park, Sangyeob Han, Chulhong Kim

**Affiliations:** 1 Future IT Innovation Laboratory, Department of Creative IT Engineering, Pohang University of Science and Technology (POSTECH), 77 Cheongam-ro, Nam-gu, Pohang, Gyeongbuk, 37673 Republic of Korea; 2 School of Interdisciplinary Bioscience and Bioengineering, Pohang University of Science and Technology (POSTECH), 77 Cheongam-ro, Nam-gu, Pohang, Gyeongbuk, 37673 Republic of Korea; 3 Oz-tec Co., Ltd., Rm 901, IT convergence industrial bldg., 47, Gyeongdaero 17 gil, Bukgu, Daegu, 41566 Republic of Korea

## Abstract

Optical-resolution photoacoustic microscopy (OR-PAM), a promising microscopic imaging technique with high ultrasound resolution and superior optical sensitivity, can provide anatomical, functional, and molecular information at scales ranging from the microvasculature to single red blood cells. In particular, real-time OR-PAM imaging with a high signal-to-noise ratio (SNR) is a prerequisite for widespread use in preclinical and clinical applications. Although several technical approaches have been pursued to simultaneously improve the imaging speed and SNR of OR-PAM, they are bulky, complex, not sensitive, and/or not actually real-time. In this paper, we demonstrate a simple and novel OR-PAM technique which is based on a typical galvanometer immersed in non-conducting liquid. Using an opto-ultrasound combiner, this OR-PAM system achieves a high SNR and fast imaging speed. It takes only 2 seconds to acquire a volumetric image with a wide field of view (FOV) of 4 × 8 mm^2^ along the X and Y axes, respectively. The measured lateral and axial resolutions are 6.0 and 37.7 μm, respectively. Finally, as a demonstration of the system’s capability, we successfully imaged the microvasculature in a mouse ear *in vivo*. Our new method will contribute substantially to the popularization and commercialization of OR-PAM in various preclinical and clinical applications.

Photoacoustic microscopy (PAM) has been spotlighted as a promising hybrid microscopic imaging technique based on optical excitation and ultrasound detection, the photoacoustic effect^1^,^2^,^3^,^4^,^5^,^6^. In particular, optical resolution-PAM (OR-PAM) provides label-free microscopic images, using a tightly focused laser beam and ultrasound detection. Thanks to the strong optical absorption of endogenous contrasts such as hemoglobin, melanin, and lipids, OR-PAM can noninvasively provide anatomical information (e.g., angiogenesis, vessel structures, melanin distribution, and nerves), functional information (e.g., hemoglobin oxygen saturation and blood flow), and metabolic contrast^7^. OR-PAM has found broad application in the biomedical and life science research fields, including ophthalmology, dermatology, neurology, oncology, and cardiology^8^,^9^,^10^,^11^,^12^. Nevertheless, to meet the demands of preclinical and clinical use, OR-PAM must achieve real-time imaging with a high signal to noise ratio (SNR). To realize a high SNR, the first and second generations of OR-PAM used an opto-ultrasound combiner with a co-focused optical and acoustic scheme^13^,^14^. Unfortunately, these microscopes suffered from slow imaging speeds caused by their mechanical linear scanning.

To increase the imaging speed and SNR, alternative OR-PAM systems tried using a one-dimensional array ultrasound transducer, a voice coil with an opto-ultrasound combiner, or a cylindrically focused transducer^15^,^16^,^17^. Although they provided relatively fast imaging compared with the previous mechanical scanning method, they were bulky and fell short of real-time imaging (B-scan rate <40 Hz/mm^18^). Recently, a compact waterproof microelectromechnical system (MEMS) scanner was applied in an OR-PAM system with an opto-ultrasound combiner. With the target immersed in water, the waterproof MEMS scanner provided simultaneous angular scanning of the laser and ultrasound, achieving fast real-time PA imaging of brain activity^19^ and flowing carbon particles^20^,^21^. By scanning the laser and ultrasound directly instead of with the use of heavy instruments, the waterproof MEMS system attained its potential maximum imaging speed. However, its performance depends on the handmade waterproof MEMS scanner, and such scanners are not widely available. In an alternative approach, a commercial optical scanner (i.e., a galvanometer) was used to provide fast and stable OR-PAM imaging^22^, but only the laser was scanned, and the unfocused ultrasound detection configuration resulted in a low SNR. Unlike the waterproof MEMS scanner, a normal galvanometer works in air, a poor conductor of sound, which makes a co-focused optical and acoustic detection scheme infeasible.

In this paper, we propose a novel high speed and high SNR galvanometer-based OR-PAM operating in a non-conducting liquid (GM-OR-PAM in NCL). The non-conducting liquid acoustic propagation medium allows using a galvanometer in combination with an opto-ultrasound combiner for simultaneous acoustic and optical scanning. To demonstrate the feasibility of GM-OR-PAM in NCL, we investigated acoustic and optical properties such as ultrasound velocity, attenuation, and optical transmittance in non-conducting liquid. We then experimentally determined the system’s field of view (FOV), imaging speed, and quantitative spatial resolution, and successfully acquired *in vivo* OR-PAM images of the microvasculature in a mouse’s ear.

## Results

### Acoustic and optical properties of the non-conducting liquid

**Figure 1:**
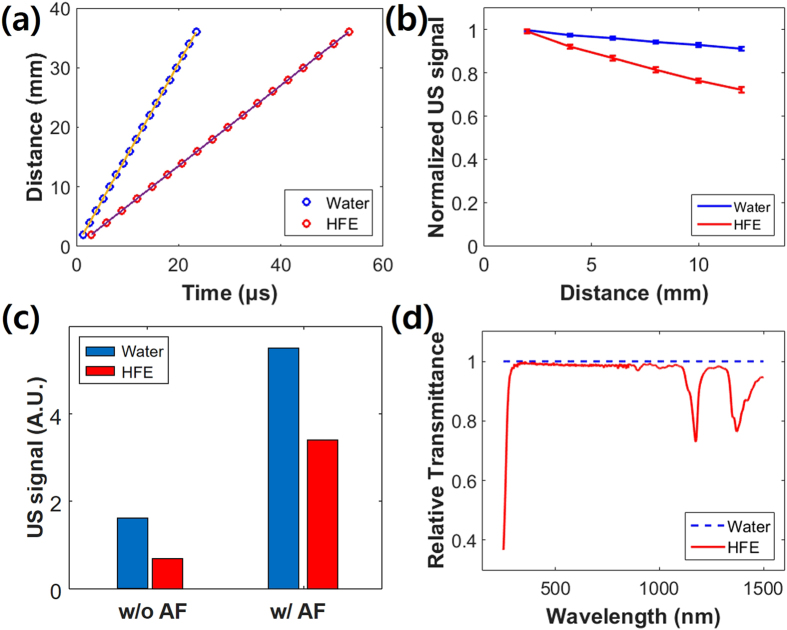
(**a**) Ultrasound velocity and (**b**) ultrasound attenuation at different distances in a non-conducting liquid (HFE) and deionized (DI) water using an unfocused transducer. (**c**) Ultrasound signals from unfocused (wo/AF) and focused (w/AF) transducers at 11 mm distance in HFE and DI water. (**d**) Relative optical transmittance of HFE compared to water. US, ultrasound; AF, acoustic focusing; HFE, hydrofluoroether.

To quantify the acoustic and optical properties of the non-conducting liquid, we measured the velocity and attenuation of ultrasound signals over different distances by propagating pulsed ultrasound in sample media. As the non-conducting liquid, we used hydrofluoroether (HFE), which is typically used as a coolant for electronic components or equipment. Deionized (DI) water was used as reference. As shown in Fig. 1(a), the measured ultrasound velocities of HFE and DI water were 676 and 1536 m/sec, respectively. That is, ultrasound propagation in HFE was about 2.3 times slower than in DI water. Using the given acoustic speed and density, the calculated acoustic impedance of HFE was 1.1 MRayls. When ultrasound travels from soft tissues (e.g., 1.6 MRayls) to HFE, the ultrasound reflection is 3.4%. Although this value is higher than that in water (0.1%), we believe that it is sufficiently low to be used as an ultrasound coupling medium. Figure 1(b) shows the attenuation profiles of ultrasound signals in HFE and water. As the propagation distance increases, the ultrasound signal was more attenuated in HFE than in DI water: The attenuation coefficients of HFE and DI water were estimated as 0.030 and 0.007 mm^−1^, respectively. For example, there was 25.7% signal attenuation in HFE at 11 mm, but only 7.9% attenuation in DI water. To address this concern, we tested a focused ultrasound configuration in HFE and DI water at a focal length of 11 mm, as shown in Fig. 1(c). The focused ultrasound signal in DI water was about three times stronger than the unfocused signal. Although the absolute ultrasound signal in HFE was weaker than that in water, the focused ultrasound signal was about five times stronger than the unfocused signal. This acoustic attenuation in HFE should be even further reduced in PAM because PAM uses the one-way trip of ultrasound not round trip. Thus, we could be confident that a focused ultrasound detection configuration would successfully compensate for the PA signal loss in HFE. To determine the optical transmittance of HFE, we measured its relative transmittance spectrum versus water. Figure 1(d) shows the relative transmittances of HFE and DI water in the range of 250–1500 nm. Like water, HFE was transparent over a wide visible region, including the 532 nm wavelength of the proposed system’s laser.

### GM-OR-PAM in NCL

**Figure 2:**
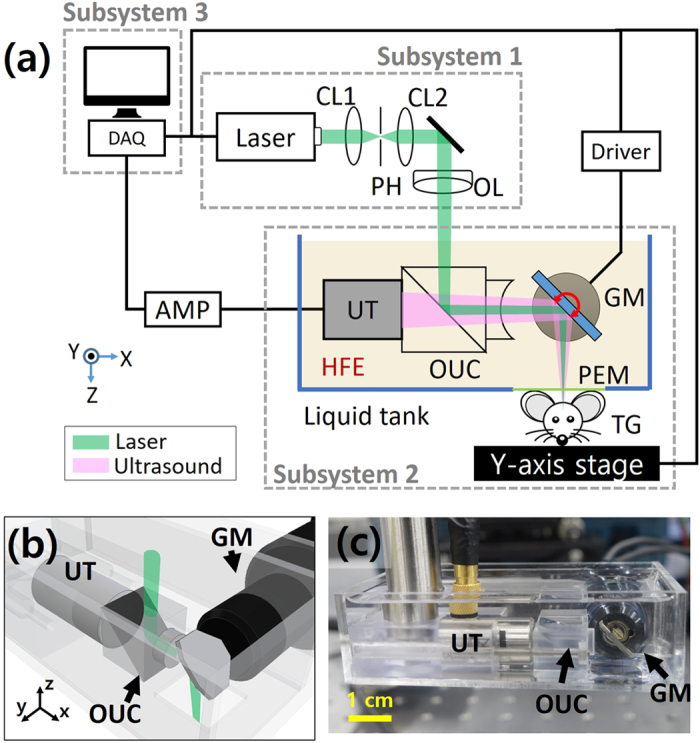
(**a**) Schematic of the galvanometer-based OR-PAM in non-conducting liquid (GM-OR-PAM in NCL). (**b**) 3D CAD design and (**c**) photograph of the GM-OR-PAM in NCL system (Supplementary Video S1). GM, galvanometer; OUC, opto-ultrasound combiner; UT, ultrasound transducer; CL, condenser lens; OL, objective lens; PH, pinhole; PEM, polyethylene membrane; TG, target.

Figure 2(a) is a schematic diagram of the GM-OR-PAM in NCL. Functionally, it consists of three main subsystems: (1) laser delivery (2) scanning and detection, and (3) PA data acquisition and processing. Detailed implementation of the GM-OR-PAM in NCL is described in the Methods section. In the laser delivery subsystem, a nanosecond pulsed laser at a 532 nm optical wavelength generates PA waves. The laser repetition rate is controlled from 10 kHz to a maximum of 50 kHz. The laser output is collimated and focused into the sample. In the scanning and detection subsystem, the opto-ultrasound combiner is implemented. After the focused laser beam is reflected from an aluminum film in between the two prisms, it is directed into the sample by a galvanometer mirror. As shown in the previous acoustic attenuation experiment using HFE, the acoustic lens focuses and amplifies the ultrasound for detection. Moreover, coaxial and confocal alignment with the focused laser, achieved by using the beam combiner, maximizes the SNR. The mirror on the galvanometer is coated with aluminum film to reflect both the laser beam and ultrasound in HFE. Thus, the co-aligned focused laser and the ultrasound beam are simultaneously scanned on the target. All these components are submerged inside a small handmade liquid container (70 mm × 40 mm × 20 mm along the X, Y, and Z axes shown in Fig. 2(b,c)). To obtain PA B-scan images, a one-dimensional galvanometer scan is performed along the X axis (Supplementary Video S1), and a linear motorized stage moves the target along the Y axis to acquire volumetric PA images. The generated PA waves are acquired by an ultrasound transducer and converted to PA images through the PA data acquisition and processing subsystem.

### FOV and imaging speed

**Figure 3:**
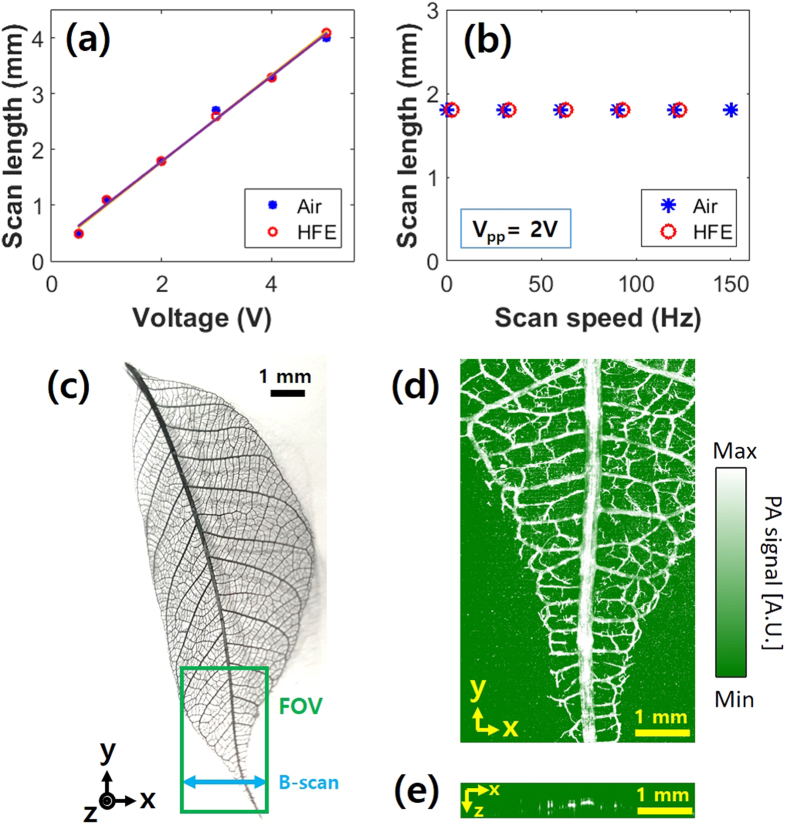
Field of view (FOV) and imaging speed of the GM-OR-PAM in NCL. Scanning length versus (**a**) the applied voltage and (**b**) scanning speed. (**c**) Photograph of a leaf skeleton and (**d**) corresponding PA maximum amplitude projection (MAP) image (Supplementary Video S2). (**e**) B-scan image. PA, photoacoustic.

In an initial experiment, we measured the FOV and imaging speed of the GM-OR-PAM in NCL by imaging a leaf skeleton target (Fig. 3(c)). The scan length along the X-axis was determined by the focal length of the acoustic lens and the scanning angle of the galvanometer. With an 11-mm-fixed-focal-length of the acoustic lens, we found the linear relationship of the scanning length versus the voltage applied to the galvanometer over a range of 0.5–4.0 mm in both air and HFE (Fig. 3(a)). Further, the scan length was independent on the scanning speed (Fig. 3(b)). However, the scanning speed of the galvanometer in HFE is limited to 150 Hz due to strong damping. The scan length along the Y-axis could be longer than that along the X-axis because it is directly determined by the <25 mm travel range of the motorized stage. Figure 3(d,e) respectively show a PA maximum amplitude projection (MAP) image of the green boxed region and a cross-sectional B-scan image along the blue arrow in Fig. 3(c). The wide FOV of 4 × 8 mm^2^ along the X and Y directions was sufficient to image this large target. The PA imaging speed is closely related to the repetition rate of the pulsed laser because one laser pulse generates one A-line PA signal along the depth direction. Since the GM-OR-PAM in NCL can fully utilize the fast scanning ability of the galvanometer, all pulses are used to form PA images, without any waste. Therefore, the B-scan and volumetric scanning speeds can be simply estimated by dividing the laser repetition rate by number of X pixels and the number of total pixels, respectively. For instance, the imaging speeds of one B-scan and one volumetric image in Fig. 3(e,d) were 250 and 0.5 Hz, respectively, with 200 × 500 pixels at the maximum A-scan rate (i.e., the repetition rate of the laser) of 50 kHz (Supplementary Video S2).

### Spatial resolution and SNR

**Figure 4:**
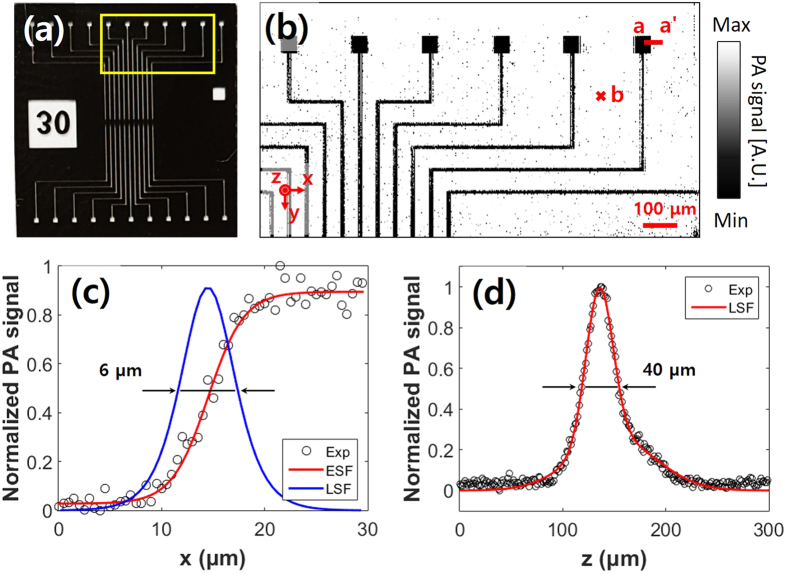
Spatial resolution of the GM-OR-PAM in NCL. (**a**) Photograph of a micro pattern. (**b**) PA MAP of the yellow line boxed area in (**a**). (**c**) Lateral resolution from fitting the ESF across line a-a’ in (**b**). (**d**) Axial resolution determined by LSF fitting along the depth direction at point ‘b’ in (**b**). ESF, edge spread function; LSF, line spread function.

In order to measure the spatial resolution and SNR of the developed system, we prepared a micro pattern as shown in Fig. 4(a), with the smallest dimension of 10 μm and a thickness of 5 μm. After acquiring a PA MAP image was acquired (Fig. 4(b)), both the lateral and axial Hilbert transformed profiles were quantified across the X-axis (line a-a’) and the Z-axis, respectively. Shown in Fig. 4(c), the lateral resolution was calculated from line spread function (LSF), which is derived from the first derivative of the edge spread function (ESF). The full width at half-maximum (FWHM) of the LSF indicates that the lateral resolution is 6.0 μm, which is close to the theoretical value of 5.1 μm. Figure 4(d) shows the LSF fitting of a randomly selected A-line at ‘b’ point in Fig. 4(b). The axial resolution was measured as 37.7 μm, while the theoretical value based on 50 MHz bandwidth of the ultrasonic transducer is 27 μm. This difference originated from the ultrasound velocity difference between the HFE and water media. PA waves were transmitted from the sample to the ultrasonic transducer through a long path in HFE. The slow transmission speed in HFE widened the pulse width of the PA wave and made the PA bandwidth narrower. Consequently, the axial resolution was worse than expected. The measured SNR in the Fig. 4(d) is 44 dB which is about 8 times higher than that of the PAM systems with optical scanning only^22^.

### *In vivo* PA imaging

**Figure 5:**
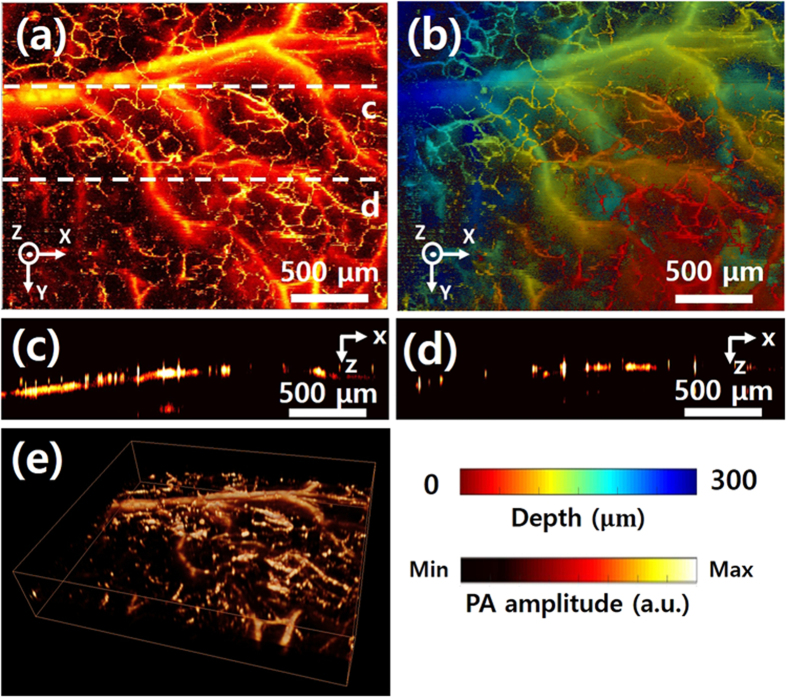
*In vivo* PA imaging of GM-OR-PAM in NCL of a mouse ear. (**a**) PA MAP image of microvasculatures of mouse ear. (**b**) Depth-encoded PA MAP image corresponding to (**a**). (**c**,**d**) B-scan cross sectional PA images of mouse ear along the white dashed lines in (**a**). (**e**) 3D PA volumetric image (Supplementary Video S3).

To demonstrate the high resolution and SNR of the GM-OR-PAM in NCL with, we conducted *in vivo* experiments in live mice. A continuous triangular waveform with a frequency of 5 Hz was applied for the galvanometer scanning along the X axis. Linear scanning along the Y axis was employed for volumetric PA imaging. The image acquisition time for 1000 × 1000 pixels was 100 seconds, and the measured FOV was 2.5 × 2 mm^2^ along the X and Y directions. Figure 5(a) shows amplitude based PA MAP images of the microvasculature of the mouse ear, and Fig. 5(b) shows the corresponding depth-encoded PA MAP image. Arteries and veins are clearly distinguished. Moreover, capillaries are clearly identifiable in both images. Figure 5(c,d) are B-scanned PA images along the white dashed lines of (a), showing the microvasculature in cross-section. As a final demonstration, a volumetric 3D image, processed by commercial software (Amira 6, FEI, USA), is shown in Fig. 5(e) (Supplementary Video S3).

## Discussion

We have demonstrated a new OR-PAM system using an easily available non-conducting liquid and a galvanometer. The high speed and sensitivity of this GM-OR-PAM in NCL are achieved without a handmade scanner. Although the tested non-conducting liquid has an inherent loss of the acoustic signal during propagation, the opto-ultrasound combiner offsets this loss and enables highly-sensitive PA imaging. The performance of GM-OR-PAM in NCL was validated by quantifying its spatial resolution and imaging speed, and by high resolution imaging of the microvasculature in a live mouse ear. We expect that this new method will further extend the applications of OR-PAM to such fields such as industrial inspection. In the future, we will focus on further improvements: (1) Applying a two axis galvanometer mirror to achieve real-time volumetric PA imaging with a more compact instrument, (2) Enhancing axial-resolution by shortening the path length in HFE or changing to another non-conducting liquid, (3) Applying feedback control methods to further enhance scanning accuracy and linearity^23^.

## Materials and Methods

### Acoustic and optical properties of HFE

As the non-conducting liquid, we purchased HFE (Novec 7500 Engineering Fluid, 3M, USA). DI water was used as reference. An unfocused transducer (V214-BB-RM, Olympus NDT, USA) with a 50 MHz center frequency was the ultrasound transmission and receiving component. Measured ultrasound signals were amplified by a pulser/receiver (5072PR, Olympus NDT, USA) and then recorded by a digitizer (ATS9350, AlazarTech, Canada). The surface of a plastic water tank formed a reflecting target. To measure optical transmittance spectrum of HFE versus water, we used a UV/Vis spectrophotometer (V-670, JASCO, USA).

### GM-OR-PAM in NCL

In the laser delivery subsystem, a nanosecond Q-switched pulsed laser at a 532 nm optical wavelength (SPOT-10-200-532, Elforlight, UK) is used. Trigger signals from a multifunctional data acquisition board (NI PCIe-6321, National Instruments, USA) initiate the pulsed laser illumination, and also synchronizes the galvanometer scanning and PA data acquisition. The laser output is collimated and spatially filtered by two achromatic lenses (LA1805 and LA1131, Thorlabs, USA) and a pinhole (P50C, Thorlabs, USA). Then, an objective lens (AC254-075-A, Thorlabs, USA) focusses the beam into the sample.

In the scanning and detection subsystem, the opto-ultrasound combiner is implemented by two prisms (NT32-330 and NT32-331, Edmund, USA) and an acoustic lens (NT45-010, Edmund, USA). To obtain PA B-scan images and volumetric PA images, a galvanometer (GVS001, Thorlabs, USA) and a linear motorized stage (PT1-Z8, Thorlabs, USA) are used along the X and Y axes, respectively. As a PA signal coupling medium, HFE fills the container. At the bottom of the container, a polyethylene membrane seals an opening and passes both laser illumination and ultrasound waves. To conduct *in vivo* animal experiments, ultrasound gel is used for signal coupling. The generated PA waves are acquired by an ultrasound transducer (V214-BB-RM, 50 MHz center frequency, Olympus NDT, USA).

In the PA data acquisition and processing subsystem, the acquired PA waves are amplified by two serially connected amplifiers (ZFL-500LN, Mini-Circuits, 52 dB gain, USA), and converted to digital signals via a digitizer. All of data processing, including Hilbert transformation and continuous display of the B-scan and volumetric images, is coded using LabVIEW software (Version 13.0, National Instruments, USA).

### Animal preparation for *in vivo* imaging experiments

All animal procedures were approved by the Institutional Animal Care and Use Committee of the Pohang University of Science and Technology (POSTECH). And all animal experiments were carried out in accordance with the National Institutes of Health Guide for the Care and Use of Experimental Animals. A normal white colored Balb/c mouse (weight ~20 g) was prepared for *in vivo* ear PA imaging. The mouse was initially anesthetized with 3% vaporized isoflurane gas, and then it was maintained with 0.75% isoflurane during PA imaging. The mouse was placed on an animal holder with a heating pad to maintain its body temperature. The pulsed laser illumination energy of 11.1 mJ/cm^2^ was within the ANSI limitation of ~ 20 mJ/cm^2^ for visible light.

## Additional Information

**How to cite this article**: Kim, J. Y. *et al*. High-speed and high-SNR photoacoustic microscopy based on a galvanometer mirror in non-conducting liquid. *Sci. Rep.* **6**, 34803; doi: 10.1038/srep34803 (2016).

## Supplementary Material

Supplementary Information

Supplementary Video S1

Supplementary Video S2

Supplementary Video S3
