# Supplementary material for: High-speed and high-SNR photoacoustic microscopy based on a galvanometer mirror in non-conducting liquid
Source: Sci Rep. 2016 Oct 6;6:34803. doi: 10.1038/srep34803 (PMC5052531; doi:10.1038/srep34803)
Supplement: Supplementary Information [file srep34803-s1.doc]

Supplementary information

High-speed and high-SNR photoacoustic microscopy based on a galvanometer mirror in non-conducting liquid

**Jin Young Kim^1^**^†^**, Changho Lee1**^†¶^**, Kyungjin Park^2^, Sangyeob Han^3^, and Chulhong Kim^1*^**

^1^Future IT Innovation Laboratory, Department of Creative IT Engineering, Pohang University of Science and Technology (POSTECH), 77 Cheongam-ro, Nam-gu, Pohang, Gyeongbuk, Republic of Korea, 37673

^2^ School of Interdisciplinary Bioscience and Bioengineering, Pohang University of Science and Technology (POSTECH), 77 Cheongam-ro, Nam-gu, Pohang, Gyeongbuk, Republic of Korea, 37673

^3^Oz-tec Co., Ltd., Rm 901, IT convergence industrial bldg., 47, Gyeongdaero 17 gil, Bukgu, Daegu, Republic of Korea, 41566

^†^These authors contributed equally to this work.

^¶^Current address: Department of Electrical and Computer Engineering, Johns Hopkins University, 3400 North Charles Street, Baltimore, MD 21218, USA

*Correspondence and requests for materials should be addressed to C.K. ([chulhong@postech.edu](mailto:chulhong@postech.edu))


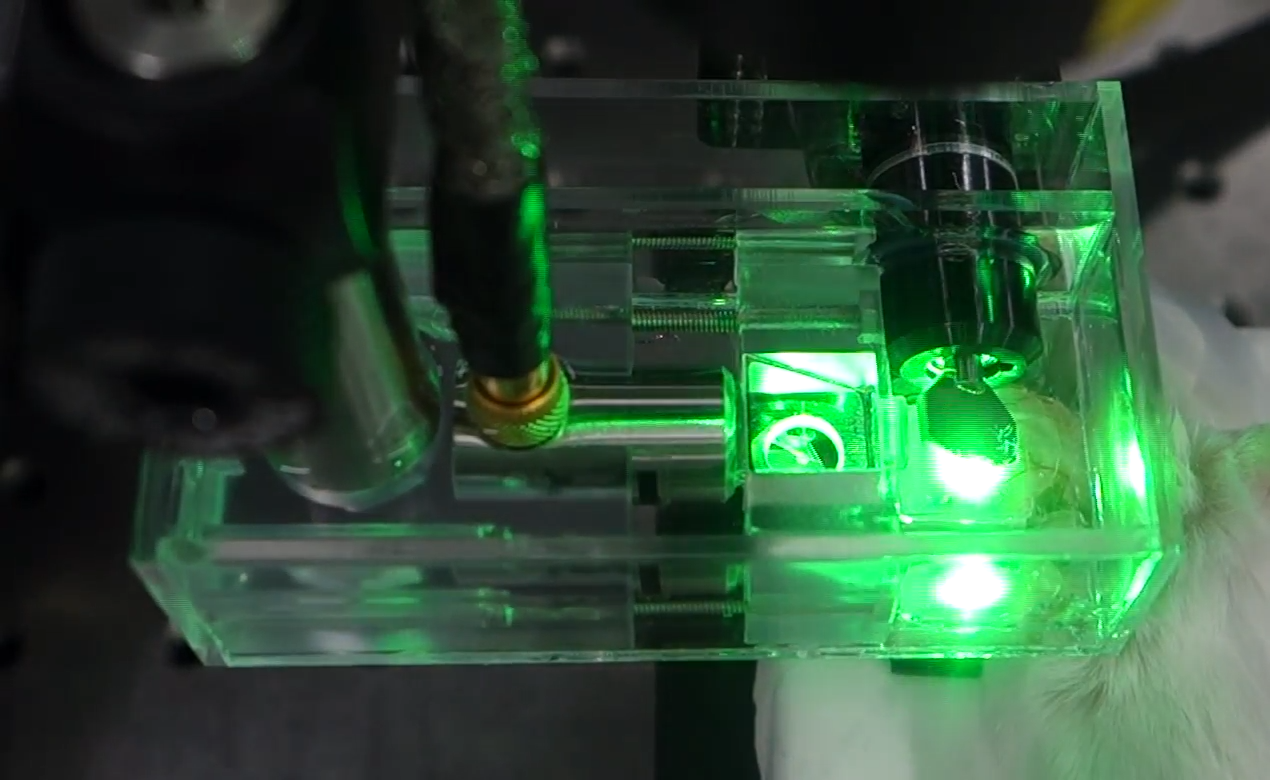


**Supplementary Video. S1**. Movie of the scanning motion of the GM-OR-PAM in NCL system.


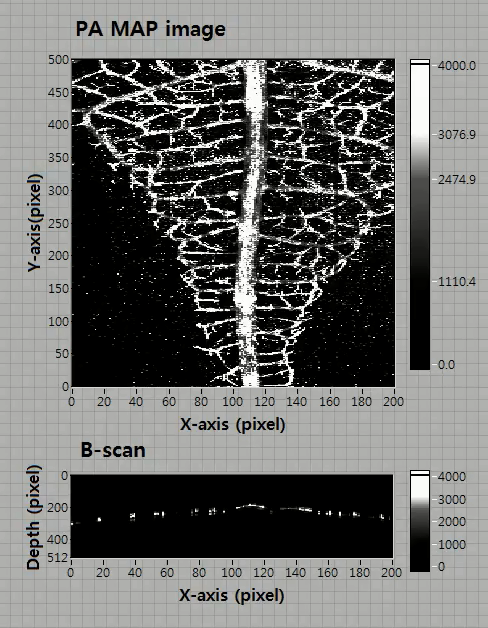


**Supplementary Video. S2**. *In vitro* PA imaging movie of the black leaf skeleton target. 500 B-scan images and corresponding one PA MAP image are displayed in 2 seconds.


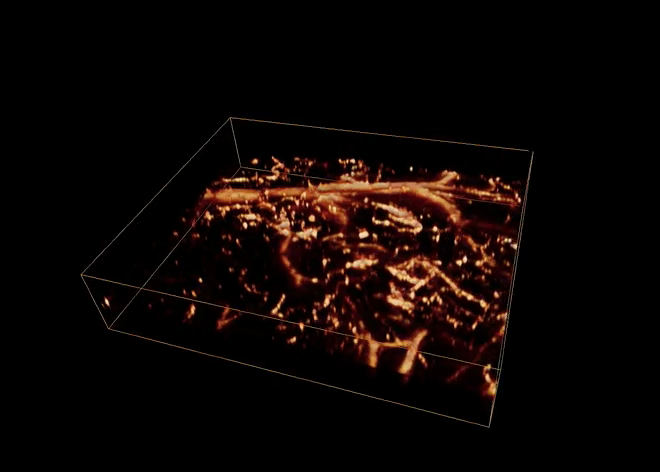


**Supplementary Video. S3**. *In vivo* 3D volumetric imaging movie of the mouse ear. The movie is processed by commercial software (Amira 6, FEI, USA).
